# Network Pharmacology-Based Investigation of the Mechanism of Action of Plantaginis Herba in Hyperuricemia Treatment

**DOI:** 10.1155/2021/5595384

**Published:** 2021-04-08

**Authors:** Rong Tang, Xiaoqing Peng, Yan Wang, Xiaohong Zhou, Hong Liu

**Affiliations:** ^1^Department of Pharmacy, Guangzhou First People's Hospital, School of Medicine, South China University of Technology, Panfu Road 1, Guangzhou 510013, Guangdong, China; ^2^Department of Traditional Chinese Medicine, The First Affiliated Hospital of Guangdong Pharmaceutical University, Gonghexiheng Street 1, Guangzhou 510080, Guangdong, China

## Abstract

This study used a network pharmacology approach to investigate the potential active ingredients of Plantaginis Herba and its underlying mechanisms in hyperuricemia treatment. The potential active ingredients of Plantaginis Herba were obtained from TCMSP and ETCM databases, and the potential targets of the active ingredients were predicted using the Swiss TargetPrediction database. The potential therapeutic targets of hyperuricemia were retrieved from the GeneCards, DisGeNET, and Online Mendelian Inheritance in Man (OMIM) databases. Then, the integrative bioinformatics analyses of candidates were performed by GO analysis, KEGG analysis, and PPI network construction. There were 15 predicted active ingredients in Plantaginis Herba and 41 common targets that may be involved in the treatment of hyperuricemia. A total of 61 GO annotations and 35 signaling pathways were identified by enrichment analysis (*P* < 0.01). The underlying mechanisms of Plantaginis Herba may be related to insulin resistance, PI3K/AKT, TNF, VEGF, AMPK, and glucagon signaling pathways. Thus, the present study provided potential and promising strategies of Plantaginis Herba for hyperuricemia treatment.

## 1. Introduction

Hyperuricemia is a syndrome involving metabolic abnormalities (increases in uric acid production and/or decreased excretion of uric acid) caused by purine metabolism disorders [1]. Excessive uric acid in blood can lead to the formation of sodium urate crystals, which are then deposited in joints, thus inducing gout [2–4].

The pathogenesis of hyperuricemia is related to a variety of factors, such as genetics, intense exercise, alcoholism, radiotherapy and chemotherapy, renal insufficiency, drugs, and metabolic syndrome [5]. In recent years, the prevalence of hyperuricemia has significantly increased and has shown a trend of younger age of onset [6, 7]. In China, the prevalence of hyperuricemia is 13.3%, indicating that it has become another common metabolic disease, such as diabetes [8].

Currently, to treat hyperuricemia, Western medicine therapeutic strategies mainly involve the inhibition of uric acid synthesis, the promotion of uric acid excretion, and the alkalinization of urine. The commonly used Western medicines are allopurinol and benzbromarone, which can cause adverse reactions in the liver, gastrointestinal tract, and on the skin [2].

Plantaginis Herba is the dried whole grass of *Plantago asiatica* L. or *Plantago depressa* Willd, which belongs to Plantaginaceae. It can help reduce fever and diuresis, dispel phlegm, and detoxify [9]. Previous studies have shown that Plantaginis Herba reduces uric acid, which may be achieved through inhibiting the activities of xanthine oxidase (XOD) and adenosine deaminase (ADA) and downregulating the mRNA expression of renal urate transporter 1 (mURAT1) [10]. However, it is difficult to compressively explain the synergism of multi-ingredient/multitarget/multipathway of traditional Chinese medicine (TCM) through single-target or single-pathway studies. In the present study, we utilized TCM network pharmacology to systematically analyze the effective active ingredients and potential targets of Plantaginis Herba to explore its possible mechanism.

## 2. Materials and Methods

### 2.1. Prediction of the Active Ingredients of Plantaginis Herba and Its Targets

The keyword “Plantaginis Herba” was searched in the Chinese medicine systems pharmacology database and analysis platform (TCMSP) (http://tcmspw.com/) and the Encyclopedia of Traditional Chinese Medicine (ETCM) database (http://www.tcmip.cn/ETCM/), and the retrieved results were screened based on the following parameters: oral bioavailability (OB) ≥ 30% and drug-likeness (DL) ≥ 0.18. Then, the existing literature was searched for studies involving the ingredients of Plantaginis Herba for hyperuricemia treatment. The molecular structures (SDF format) of the obtained ingredients were obtained from the PubChem database (https://pubchem.ncbi.nlm.nih.gov/), and potential targets were predicted using the Swiss TargetPrediction database (http://www.swisstargetprediction.ch/). The potential targets were subjected to gene normalization using the UniProt database (http://www.UniProt.org/). The active ingredients and corresponding targets of Plantaginis Herba were imported into Cytoscape 3.8.0 software to construct an active ingredient-target network for Plantaginis Herba.

### 2.2. Prediction of Hyperuricemia-Related Targets

“Hyperuricemia” and “hyperuricaemia” were used as keywords to search the following disease gene databases: DrugBank (https://www.drugbank.ca/), DisGeNET (https://www.disgenet.org/), GeneCards (https://www.genecards.org/), Online Mendelian Inheritance in Man (OMIM) (https://www.omim.org/), and Therapeutic Target Database (TTD) (http://db.idrblab.net/ttd/). Additionally, using the data retrieved from DrugBank, targets of first-line Western drugs used for the clinical treatment of hyperuricemia were also included. The obtained target genes were imported into the UniProt database for gene standardization.

### 2.3. Protein-Protein Interaction (PPI) Network Analysis

The targets of the active ingredients of Plantaginis Herba and hyperuricemia-related targets were imported into Venny 2.1 software to generate a Venn diagram to obtain the common targets of Plantaginis Herba for the treatment of hyperuricemia. The common targets were imported into the STRING database (https://string-db.org/), and the species “*Homo sapiens*” was selected to obtain a PPI network. The data were then imported into Cytoscape 3.8.0 software for visualization, and the core targets in the PPI network were obtained.

### 2.4. GO Analysis and KEGG Pathway Enrichment Analysis

The overlapped targets were imported into DAVID 6.8 (https://david.ncifcrf.gov/) for Gene Ontology (GO) analysis and Kyoto Genomics and Genomics Encyclopedia (KEGG) analysis; the screening parameter was set as the false discovery rate (FDR) < 0.05. The results were visualized using Image GP, and the analyzed results were displayed using bubble diagrams.

### 2.5. Construction of the Target-Pathway Network

Targets enriched in the pathways associated with hyperuricemia were ascertained, and the active ingredients acting on these targets were identified. The interaction among the active ingredients, targets, and pathways was established using nodes to represent the active ingredients, targets, and pathways and edges to represent the association between a certain target and a certain pathway or the action of an active ingredient on a certain target. The file data were imported into Cytoscape 3.8.0 to construct an “active ingredient-target-pathway” network, and the topological attributes of the nodes in this network were analyzed to clarify the material basis and multi-ingredient, multitarget, and multipathway molecular mechanisms for the treatment of hyperuricemia with Plantaginis Herba.

## 3. Results

### 3.1. The Candidate Ingredients of Plantaginis Herba and Its Targets

**Figure 1:**
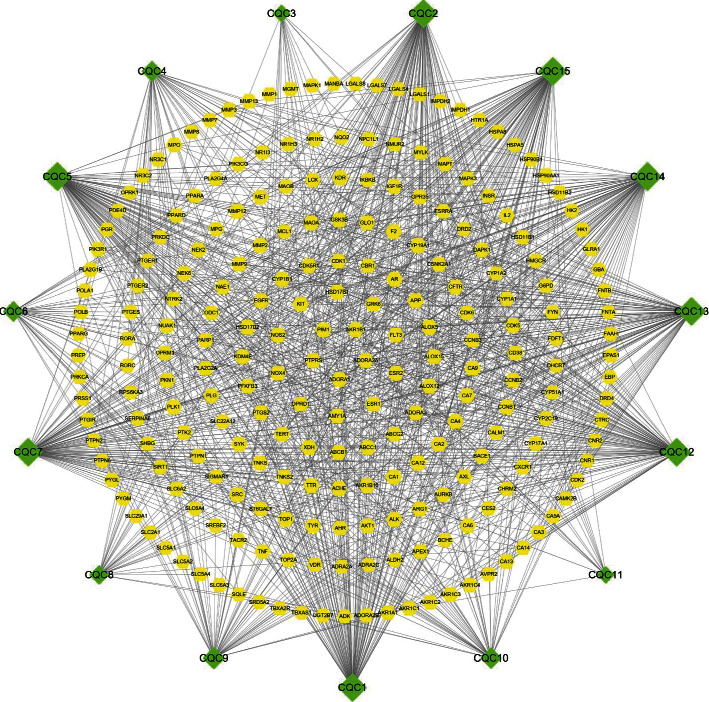
The active ingredient-target network for Plantaginis Herba. The rhombuses represent ingredient nodes, the hexagons represent predicted targets, and the connecting lines represent the interaction between the ingredients and the targets. The node size represents the corresponding degree value; a larger degree value corresponds to a larger node area, indicating that the node is more useful in the network.

We firstly screened the active ingredients of Plantaginis Herba and predicted their relative targets. Based on the OB and DL parameters, 10 active ingredients of Plantaginis Herba were obtained from the TCMSP and ETCM databases. In addition, 5 reported active ingredients of Plantaginis Herba, such as verbascoside, eupatilin, nepetin, jaceosidin, and eupatorin, were also included [11, 12]. Therefore, a total of 15 active ingredients were finally obtained (Table 1). A total of 232 targets were obtained by importing the identified active ingredients into the PubChem website and the Swiss TargetPrediction database. Cytoscape 3.8.0 software was used to visualize and analyze the networks for 15 active ingredients and 232 targets of Plantaginis Herba, and the following core ingredients were identified: hispidulin, baicalein, luteolin, 6-hydroxyluteolin, eupatilin, nepetin, jaceosidin, and eupatorin (Figure 1).

### 3.2. Construction of the PPI Network of the Active Ingredients of Plantaginis Herba and Hyperuricemia

**Figure 2:**
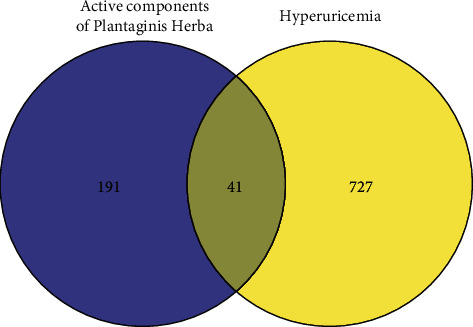
Venn diagram of targets of active ingredients of Plantaginis Herba and targets related to hyperuricemia.

**Figure 3:**
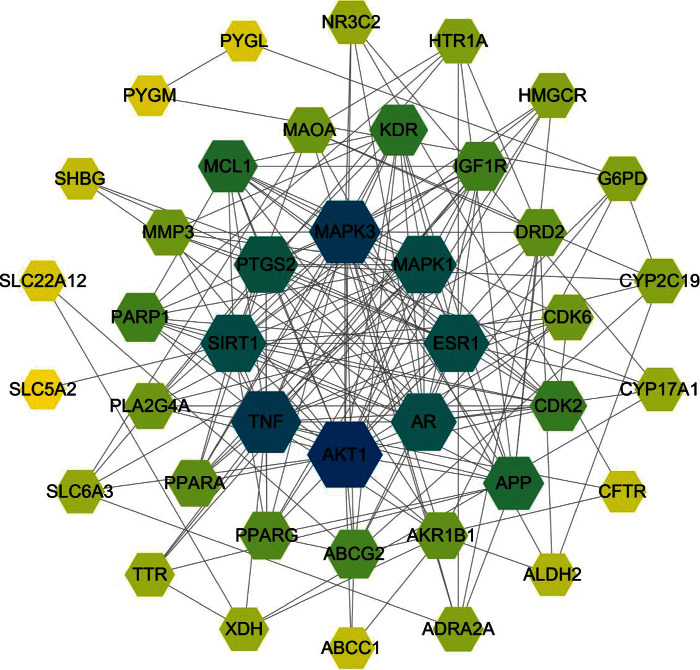
PPI network of the common targets of active ingredients of Plantaginis Herba and hyperuricemia.

Hyperuricemia-related targets were searched and retrieved from 5 disease databases: DrugBank, DisGeNET, GeneCards, OMIM, and TTD; 65, 196, 668, 6, and 0 targets were retrieved, respectively. After assessing the above targets and removing duplicate targets, we finally obtained 768 disease targets. Forty-one common targets were obtained by comparing the targets obtained above (Figure 2). These common targets were imputed into STRING to construct a PPI network, and the network was visualized using Cytoscape 3.8.0 (Figure 3). The PPI network consisted of 41 nodes with 188 edges, with an average nodal degree of 9.17 and an average compactness value of 0.584. Targets with values not less than the average values of node degree and compactness were used as core targets. The core targets of Plantaginis Herba for the treatment of hyperuricemia were AKT1, mitogen-activated protein kinase 3 (MAPK3), MAPK1, tumor necrosis factor (TNF), prostaglandin-endoperoxide synthase 2 (PTGS2), sirtuin 1 (SIRT1), estrogen receptor 1 (ESR1), and androgen receptor (AR).

### 3.3. GO Analysis and KEGG Pathway Enrichment Analysis of Hub Targets

**Figure 4:**
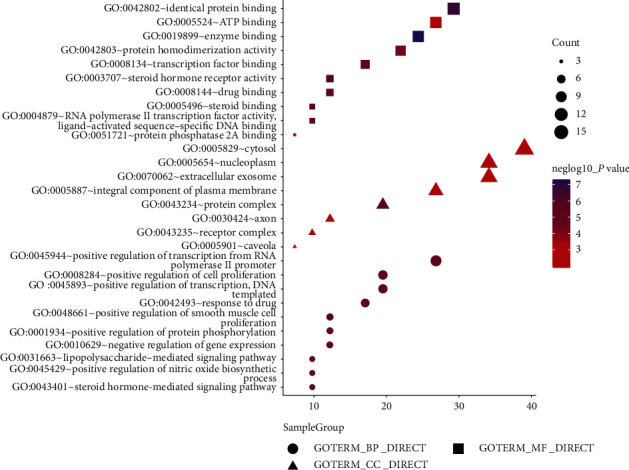
Bubble diagram of GO enrichment of common targets.

**Figure 5:**
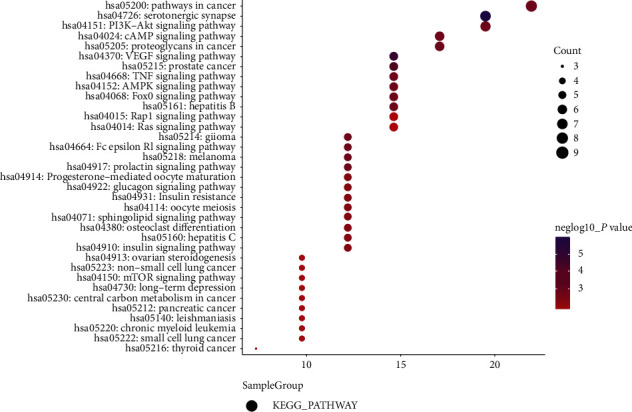
KEGG pathway analysis of common targets.

The common targets were enriched in 61 GO functions (*P* < 0.01, Figure 4). Enrichment was observed in the following molecular functions (*n* = 16): enzyme binding, protein binding, steroid receptor activity, transcription factor binding, ATP binding, and RNA polymerase II transcription factor activity. Enrichment was observed in the following biological processes: the positive regulation of RNA polymerase II promoter transcription, smooth muscle cell proliferation, positive regulation of drug reactions, nitric oxide biosynthesis, cell proliferation, negative regulation of gene expression, lipopolysaccharide-mediated signaling pathways, and steroid-mediated signaling pathways. A total of 35 KEGG pathways were enriched (*P* < 0.01, Figure 5). Among those enriched, the signaling pathways associated with hyperuricemia include the PI3K/AKT, TNF, VEGF, AMPK, insulin resistance, and glucagon signaling pathways, suggesting that Plantaginis Herba may play a role in treating hyperuricemia mainly through the regulation of these 6 signaling pathways.

### 3.4. Active Ingredient-Target-Pathway Network Analysis

**Figure 6:**
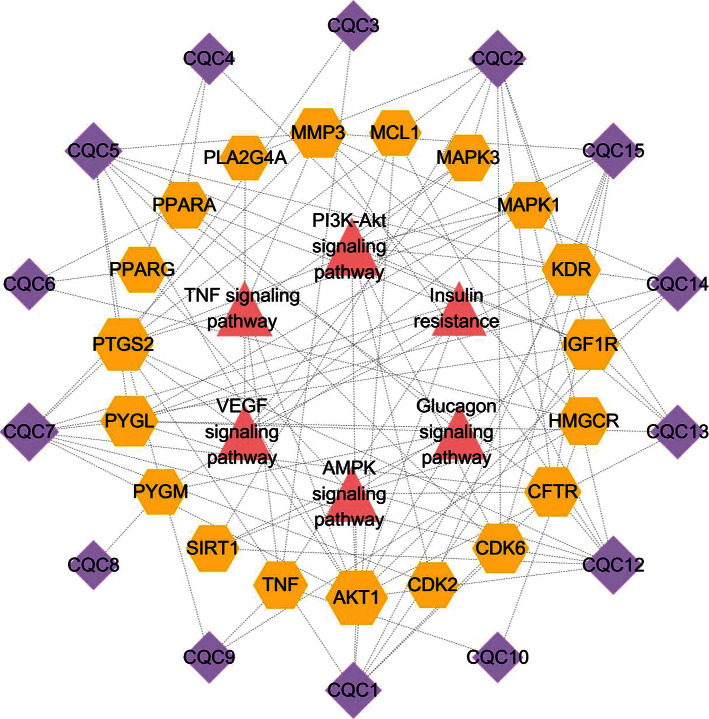
Active ingredient-target-pathway network. A triangle represents a signaling pathway, a hexagon represents a target, and a diamond represents an active ingredient. A node with a larger size indicates having more connected compounds or targets or pathways.

An “active ingredient-target-pathway” network was constructed based on the 6 most relevant signaling pathways obtained in Section 2.4 (Figure 6). There were 39 nodes (including 14 active ingredients, 19 targets, and 6 pathways) and 108 edges in the network. In this network, the active ingredients with more targets were baicalein, luteolin, eupatilin, and 6-hydroxyluteolin, suggesting that these ingredients may be the material basis through which Plantaginis Herba treats hyperuricemia. PTGS2, AKT1, IGF1R, TNF, KDR, PYGL, MAPK3, and MAPK1 were the targets that connected with more active ingredients and pathways, suggesting that these targets might be the key targets for the treatment of hyperuricemia with Plantaginis Herba; these results are consistent with the PPI network analysis results in Section 2.3. Therefore, active ingredients such as luteolin, eupatilin, 6-hydroxyluteolin, hispidulin, and baicalein act through targets such as PTGS2, AKT1, IGF1R, TNF, KDR, PYGL, MAPK3, and MAPK1 to jointly regulate signaling pathways such as PI3K/AKT, TNF, VEGF, AMPK, insulin resistance, and glucagon pathways to achieve hyperuricemia treatment efficacy.

## 4. Discussion

Plantaginis Herba clears heat, disinhibits dampness, increases diuresis, frees stranguries, eliminates phlegm, cools blood, and detoxifies [13, 14]. Modern pharmacological studies have also confirmed that the Plantaginis Herba extract can effectively treat hyperuricemia [15]. Previous studies have shown that a Chinese herbal compound containing *S. moellendorffii*, *Smilacis glabrae* Rhizoma, and Plantaginis Semen, at a ratio of 3 : 1 : 1, can significantly inhibit the activation of nuclear factor-*κ*B (NF-*κ*B) and the expression of its target genes, interleukin-1*β* (IL-1*β*), prostaglandin E2 (PGE2), and IL-8, thereby playing an important role in the prevention and treatment of hyperuricemia and gout [16]. However, the effective and important active ingredients in this compound are not known. Other studies showed that verbascoside and 1-hydroxyindole-3-carbaldehyde, the extracts of Plantaginis Herba, significantly inhibited XOD activity and had significant therapeutic effects when used to treat hyperuricemia and gout [17]. In addition, active ingredients such as baicalein, baicalin, sitosterol, 6-hydroxyluteolin, stigmasterol, luteolin, and nepetin extracted from other herbs have been shown to significantly reduce uric acid levels [17–23]. In the present study, 15 active ingredients of Plantaginis Herba were obtained from the TCMSP and ETCM databases. However, the roles of hispidulin, melampyroside, stigmasterol palmitate, *β*-sitosterol palmitate, eupatilin, jaceosidin, and eupatorin in the treatment of hyperuricemia are still unclear. Therefore, the active ingredients of Plantaginis Herba identified in this study may play important roles in the prevention and treatment of hyperuricemia.

We constructed an “active ingredient-target-pathway” network to further investigate the mechanism of action underlying Plantaginis Herba and its active ingredients in the prevention and treatment of hyperuricemia. AKT1, MAPK3 (ERK1), MAPK1 (ERK2), TNF, PTGS2 (COX-2), KDR, PYGL, and IGF1R were key targets based on a topological data analysis of the network.

Accumulating previous studies have shown that AKT plays an important role in the occurrence and development of hyperuricemia and gout. Activation of the AMP-activated protein kinase (AMPK)/AKT/cAMP-response element-binding protein (CREB) signaling axis promotes the binding of CREB to the ATP-binding cassette superfamily G member 2 (ABCG2) promoter and upregulates the expression of ABCG2, thereby promoting the excretion of uric acid [24]. Uric acid can downregulate the expression of ABCG2 by inhibiting the activation of AKT, leading to the intracellular accumulation of uric acid [25]. Interestingly, soluble uric acid activates the toll-like receptor 4 (TLR4)-NLR family pyrin domain containing 3 (NLRP3) and PI3K/AKT pathways and promotes the transcription of ABCG2 by upregulating PDZ domain containing 1 (PDZK1), thereby promoting the excretion of uric acid in the intestines, along with feces [26]. Other studies have shown that uric acid promotes the abnormal proliferation of glomerular mesangial cells through the activation of the nicotinamide adenine dinucleotide phosphate (NADPH)/ROS/extracellular receptor kinase 1/2 (ERK1/2) pathway, leading to glomerular injury [27]. Uric acid can upregulate the expression of TNF-*α* through the ROS/MAPK (p38)/NF-*κ*B signaling axis, thereby promoting the inflammation and necrosis of vascular smooth muscle cells [28]. Uric acid promotes the activation of platelet-derived growth factor receptor-*β* (PDGFR-*β*) through the p38 MAPK and ERK1/2 pathways, thereby inducing and aggravating cardiovascular diseases [29]. In a hyperuricemia animal model, uric acid can upregulate the expression of inflammatory factors such as cyclooxygenase-2 (COX-2), IL-1*β*, and TNF-*α* and play an important role in the development of complications related to hyperuricemia [30].

Other studies have shown that plantamajoside, an extract of Plantaginis Herba, can inhibit the activation of histone deacetylase 2 (HDAC2), and the transduction of downstream signals, AKT/GSK-3*β*, in turn has an important protective effect against cardiac hypertrophy [31]. Ethanol extracts of Plantaginis Herba inhibit AKT phosphorylation but can alleviate the deterioration of diabetic retinopathy through the inhibition of inflammatory signals [32]. The regulatory effects of the active ingredients of Plantaginis Herba selected in this study on AKT and their roles in the prevention and treatment of hyperuricemia and gout are still unclear and have not been reported. In addition, few studies have reported the regulatory effects of the active ingredients selected in this study on other target genes. Therefore, it is particularly important to further study the exact mechanism of action of these active ingredients.

In summary, this study selected a group of active ingredients of Plantaginis Herba with potential application prospects. They may promote ABCG2 expression and uric acid excretion through the activation of AKT-related pathways. Additionally, these active ingredients may also downregulate the body's inflammatory response by inhibiting the expression of the inflammatory factors COX-2 and TNF-*α* and inhibiting the activation of ERK1/2 and the abnormal proliferation of glomerular mesangial cells. These active ingredients may alleviate hyperuricemia, slow the occurrence and development of gout, and inhibit the damage caused by high uric acid levels in the kidney and cardiovascular system through the integration of multiple signals. Thus, the present study suggests a potential mechanism and potential clinical application values of Plantaginis Herba for the treatment of hyperuricemia and its complications.

## Figures and Tables

**Table 1:** Active ingredients of Plantaginis Herba.

No.	Mol ID	Mol name	ID	OB (%)	DL
1	MOL001735	Dinatin	CQC1	30.97	0.27
2	MOL002714	Baicalein	CQC2	33.52	0.21
3	MOL002776	Baicalin	CQC3	40.12	0.75
4	MOL000359	Sitosterol	CQC4	36.91	0.75
5	MOL004004	6-OH-Luteolin	CQC5	46.93	0.28
6	MOL000449	Stigmasterol	CQC6	43.83	0.76
7	MOL000006	Luteolin	CQC7	36.16	0.25
8	MOL007783	Melampyroside	CQC8	57.5	0.8
9	MOL007796	Stigmasterol palmitate	CQC9	38.09	0.4
10	MOL007799	*β*-Sitosterol palmitate	CQC10	30.91	0.4
11	MOL003333	Acteoside	CQC11	2.94	0.62
12	MOL005734	Eupatilin	CQC12	29.39	0.38
13	MOL005305	Nepetin	CQC13	26.75	0.31
14	MOL009297	Jaceosidin	CQC14	2.14	0.34
15	MOL001733	Eupatorin	CQC15	30.23	0.37

## Data Availability

The data used to support the findings of this study are available from the corresponding author upon request.
